# Conventional analysis of trial-by-trial adaptation is biased: Empirical and theoretical support using a Bayesian estimator

**DOI:** 10.1371/journal.pcbi.1006501

**Published:** 2018-12-26

**Authors:** Daniel Blustein, Ahmed Shehata, Kevin Englehart, Jonathon Sensinger

**Affiliations:** 1 Institute of Biomedical Engineering, University of New Brunswick, Fredericton, New Brunswick, Canada; 2 Department of Medicine, Faculty of Medicine and Dentistry, University of Alberta, Edmonton, Alberta, Canada; 3 Department of Electrical and Computer Engineering, University of New Brunswick, Fredericton, New Brunswick, Canada; Harvard University, UNITED STATES

## Abstract

Research on human motor adaptation has often focused on how people adapt to self-generated or externally-influenced errors. *Trial-by-trial adaptation* is a person’s response to self-generated errors. Externally-influenced errors applied as catch-trial perturbations are used to calculate a person’s *perturbation adaptation* rate. Although these adaptation rates are sometimes compared to one another, we show through simulation and empirical data that the two metrics are distinct. We demonstrate that the trial-by-trial adaptation rate, often calculated as a coefficient in a linear regression, is biased under typical conditions. We tested 12 able-bodied subjects moving a cursor on a screen using a computer mouse. Statistically different adaptation rates arise when sub-sets of trials from different phases of learning are analyzed from within a sequence of movement results. We propose a new approach to identify when a person’s learning has stabilized in order to identify steady-state movement trials from which to calculate a more reliable trial-by-trial adaptation rate. Using a Bayesian model of human movement, we show that this analysis approach is more consistent and provides a more confident estimate than alternative approaches. Constraining analyses to steady-state conditions will allow researchers to better decouple the multiple concurrent learning processes that occur while a person makes goal-directed movements. Streamlining this analysis may help broaden the impact of motor adaptation studies, perhaps even enhancing their clinical usefulness.

## Author summary

By observing the learning rate of a person making a movement under new conditions, researchers can better understand how the nervous system handles uncertainty. Patients suffering from motor deficits or using prostheses will often display different motor abilities that can be observed as changes in error correction rates. Here we show that previous approaches to determining error correction rates are affected by the overall learning rate within the subset of trials selected for analysis. We use real-world data collected from people controlling a computer cursor and simulations of how a person’s nervous system operates to show the limitations of current approaches. We also present a new approach to limit some of the biases with current motor analysis techniques.

## Introduction

The way that people adapt their movements provides important insight into the motor learning processes of the brain [1–4]. Motor learning, and more specifically motor adaptation, is a key aspect in the study of human movement, particularly in understanding its deficit and developing corresponding rehabilitative strategies [5,6]. *Adaptation rate* is often discussed in the literature, but that metric’s definition varies based on the context. Here we focus on trial-by-trial adaptation, rather than block adaptation such as that quantified as the time constant of an exponential fit to an individual’s movement data [1]. Trial-by-trial adaptation rates reflect how the error on a given movement affects the proportion of error correction on the following movement. Adaptation rates are often cited [2, 7–11] but are difficult to compare across conditions and studies due to differences in quantities measured, movement amplitude, system noise, and calculation methods.

A common approach to calculating dimensionless trial-by-trial adaptation rates is to run a linear regression comparing data from sequential movement trials [2,8,9]. These unit-free values were thought to be unbiased and independent of error magnitude, observations supported by state estimation Bayesian models [5]. In the absence of perturbations, adaptation to self-generated error is often calculated as the first-order regression coefficient of the change in error on the next trial versus the error on the current trial [7]. Adaptation to catch trial perturbations is calculated similarly using the first-order regression coefficient of the movement deviation (or error) versus the perturbation level [2,8,9]. These widely accepted approaches output a result that has been variably described as the ‘learning rate’ [10], ‘adaptation rate’ [11], ‘adaptation gain’ [12], or ‘adaptation coefficient’ [3].

Using a linear regression to quantify trial-by-trial adaptation, however, is not without limitations. To our knowledge there have been limited efforts to validate linear regression adaptation rates with motor control models. The analysis does not capture the effect of correlated errors beyond adjacent trials [4,12,13]. Further, reported adaptation rates represent the fusion of multiple interacting learning processes, such as explicit and implicit learning [14,15], or fast and slow processes [4,12]. In this work we will show that parameter estimation, often prominent during initial motor adaptation trials, results in a biased regression coefficient. Thus, the reporting of adaptation rates needs to be constrained and qualified; guidelines that we develop here to identify when parameter estimation has stabilized.

Advances in Bayesian modeling have given us a better understanding of the underlying organizational principles driving human motor behavior [16,17]. Non-adapting state-space-only estimators have been used to quantify adaptation [18–21] but these models may be too simple for rehabilitation contexts to fully capture the performance dynamics of the human nervous system. While learning a new task, a human will update estimates of the system’s parameters such as the mass being moved or environmental parameters like wind, gravity, visual rotation or a force field in order to improve motor planning and performance [22,23]. Here we adopt a hierarchical Kalman filter model that adds a parameter estimation filter to the state estimator in a motor control framework [5,17,24]. We use this model to simulate motor learning processes and to further explore approaches to adaptation rate analysis.

In this study we use computational modeling results and human motor data to develop a standardized approach to calculating bias-free adaptation rates. We first establish that trial-by-trial adaptation rates and perturbation adaptation rates represent fundamentally different metrics and cannot be directly compared. Then we demonstrate that trial-by-trial adaptation rates are confounded by overall learning rates. We develop an expanding window optimization approach to identify when the overall learning rate has stabilized in order to calculate an unbiased trial-by-trial adaptation rate. This steady-state trial analysis protocol is shown to produce adaptation rates that are more consistent and with less uncertainty than analyses of alternative trials sets. This computational and empirical study provides a clear path towards developing a more reliable and meaningful way to quantify human motor performance that could improve clinical motor assessments.

## Results

**Fig 1:**
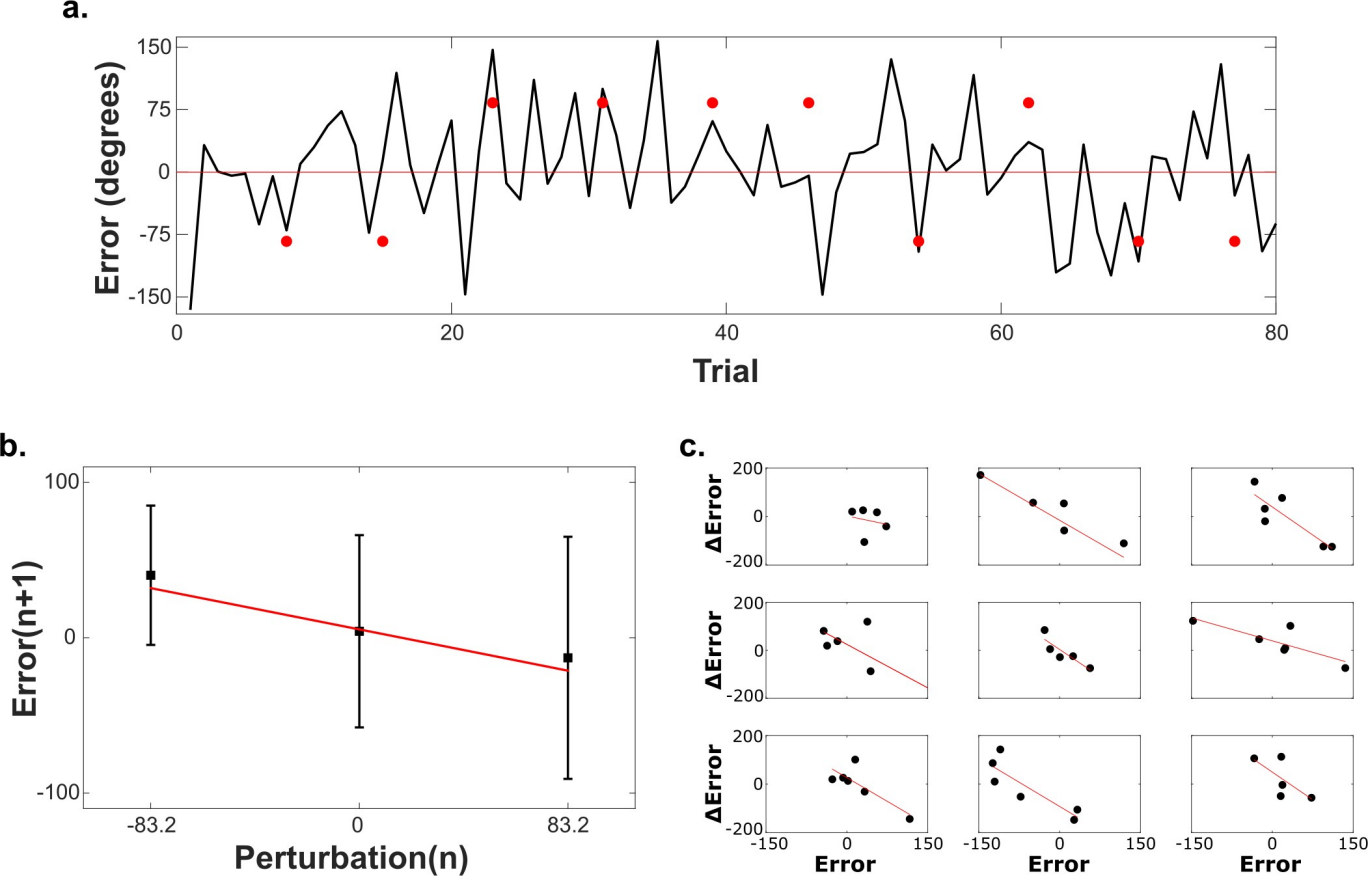
Single subject movement data with analysis of perturbation adaptation and trial-by-trial adaptation rates. (a) Trial-by-trial angular error data for a block of 80 consecutive movements of a human subject controlling a cursor with electromyographic signals. Red points indicate the trial and magnitude of catch-trial perturbations. Data from Subject ID# 2016–127 from Shehata et al. [25]. (b) Linear regression of Error_n+1_vs. Error_n_ for trials in a. Red line indicates line of best fit whose slope (-0.32) is defined as the perturbation adaptation rate. (c) Linear regression of ΔError vs. Error for each consecutive sequence of unperturbed trials between perturbations. The trial-by-trial adaptation rate (-1.20) is calculated as the mean of all regression coefficients.

Learning processes are often observed by measuring adaptation to catch-trial perturbations (Fig 1A). Without forewarning during an experiment, a person’s sequential movements are perturbed on intermittent trials. The perturbation adaptation rate is calculated as the first-order coefficient of the linear regression of error on the next trial versus perturbation on the current trial (Fig 1B). This contrasts with the definition of trial-by-trial adaptation that is represented by the first-order coefficient of the change in error to the next trial versus the error on the current trial. Typically, trial-by-trial adaptation is completed on unperturbed datasets, but for direct comparison to Fig 1B, we compute this regression for each set of unperturbed trials (Fig 1C). Each perturbation effectively resets the parameter estimation, so the short trial blocks analyzed capture some of the dynamic learning processes observed during initial trial exposure. To improve clarity, all adaptation rates in this study are reported as inverted values (i.e. negative coefficients are reported as positive adaptation rates).

**Fig 2:**
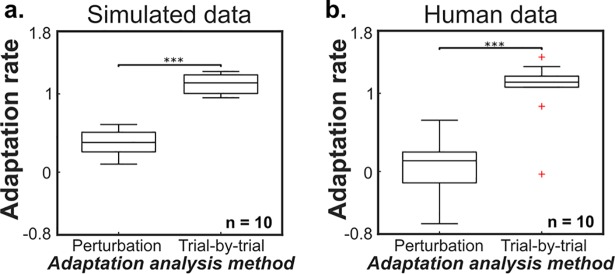
Perturbation adaptation and trial-by-trial adaptation rates are different. (a) Simulated data generated using a Bayesian learner model shows a significant difference between predicted perturbation adaptation rate and trial-by-trial adaptation rate (*** = p<0.001, two-tailed paired t-test). (b) Empirical results from 10 human subjects [25] show the same significant difference between adaptation rate analysis results (*** = p<0.001, two-tailed paired t-test).

The analysis methods of the two adaptation rates produce different results when run on the same movement data. Results from a Bayesian hierarchical Kalman filter model of human motor adaptation show that trial-by-trial adaptation rates are significantly higher than perturbation adaptation rates (Fig 2A, t-test: p<0.001, two-tailed paired t-test). These simulation results were qualitatively matched by results obtained analyzing human movement data (Fig 2B, p<0.001, two-tailed paired t-test) [25].

Under steady-state conditions, the trial-by-trial adaptation rates and perturbation adaptation rates approach different values. We ran our simulation with extreme noise parameters (Q>5, R = 0) so that the Kalman gain of the state-estimation filter approached one and the Kalman gain of the parameter-estimation filter approached zero. Under these extreme conditions, the trial-by-trial adaptation rate in unperturbed trials approached a value of one. The perturbation adaptation rate of movement trials with catch-trial perturbations approached a value of zero (regardless of perturbation frequency). The distinct results of the two adaptation rate calculation methods at simulated steady state show that they are not capturing the same quantitative measure. The two resulting adaptation rates cannot be directly compared, as has been done previously [5].

For the remainder of this study we shift to focus solely on trial-by-trial adaptation rates, an experimental paradigm that shows promise for clinical use [26] if the interaction of multiple learning processes can be disentangled [4]. The lag-1 autocorrelation coefficient has also been used to quantify human motor performance [27–29]. Since the trial-by-trial adaptation analysis and lag-1 autocorrelation results are mathematically-related (S1 Appendix), we focus only on trial-by-trial adaptation rates in this study.

**Fig 3:**
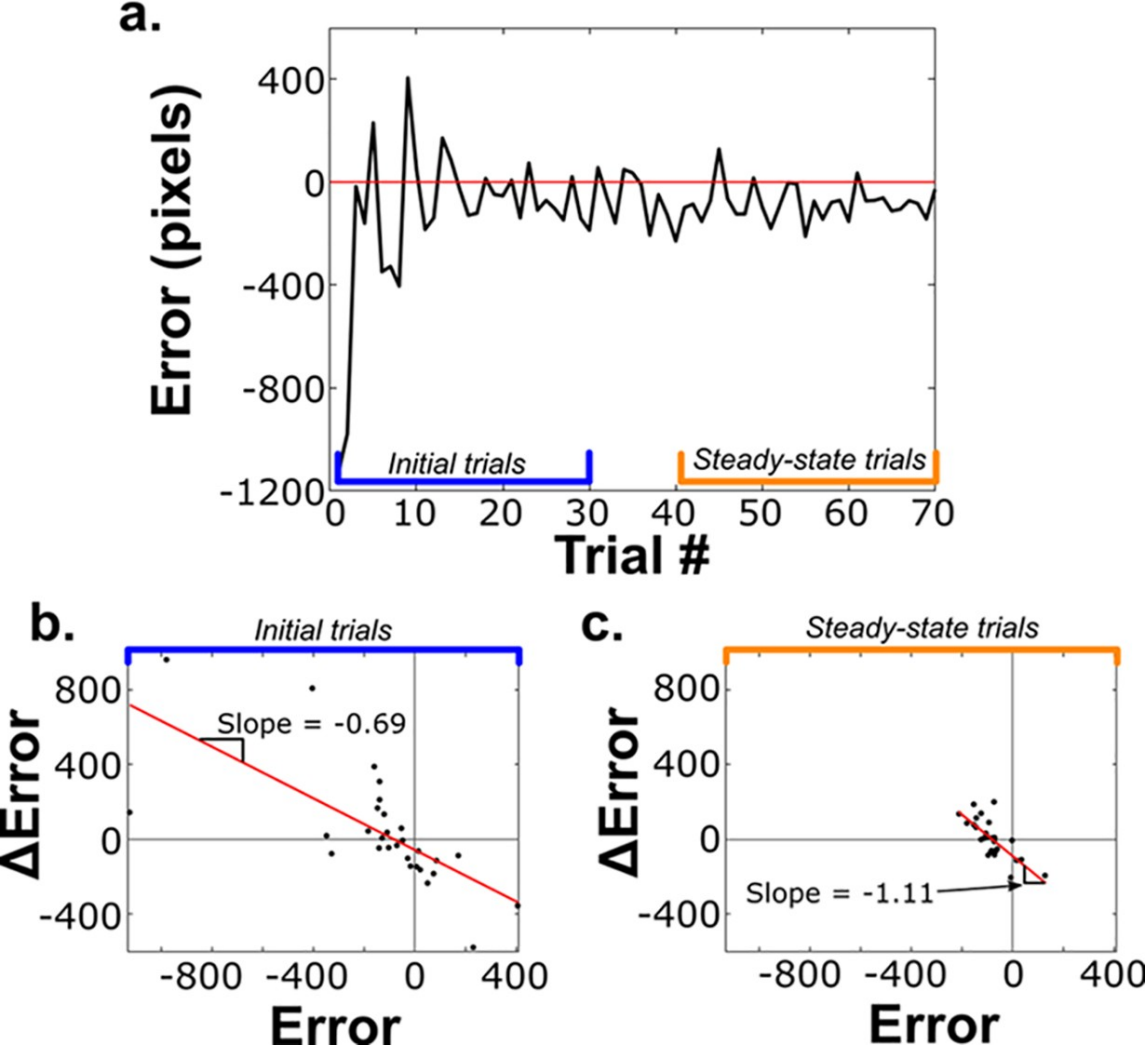
Single subject movement data and adaptation rate quantification. (a) Trial-by-trial endpoint error data (one degree-of-freedom) for a block of 70 consecutive movements of a human subject controlling an on-screen cursor with a computer mouse. (b) Linear regression of ΔError vs. Error for initial trials in a. Red line indicates line of best fit whose slope (-0.69) is defined as the adaptation rate. ΔError = Error_i+1_-Error_i_. (c) Linear regression of ΔError vs. Error for steady-state trials in a. The regression coefficient equals -1.11.

Trial-by-trial adaptation is often analyzed using a record of movement errors from a sequential set of movements without catch-trial perturbations [5,27] (Fig 3A). During a standard adaptation study, a typical set of movement data of sequential trials consists of two phases: 1. the *initial trial phase* when errors are high and parameter estimation errors are correspondingly high, and 2. the *steady-state phase* when μ(error) ≈ 0 and parameter estimates approach actual system parameters (Fig 3). As in Fig 1C, trial-by-trial adaptation rates are calculated as the first-order coefficient of a linear regression of the relationship between the change in error and error [5] (see Methods; Fig 3B and 3C).

We collected empirical movement data from 12 human subjects (one individual’s data shown in Fig 3). Participants moved a cursor on a screen, constrained along a horizontal line, using a computer mouse to hit a target. End-point only position feedback was provided to prevent feedback-driven online corrections and sub-movements, and reduce the influence of implicit learning processes [14]. Participants completed a sequence of 70 movement trials and were asked to try to hit the target with each movement.

**Fig 4:**
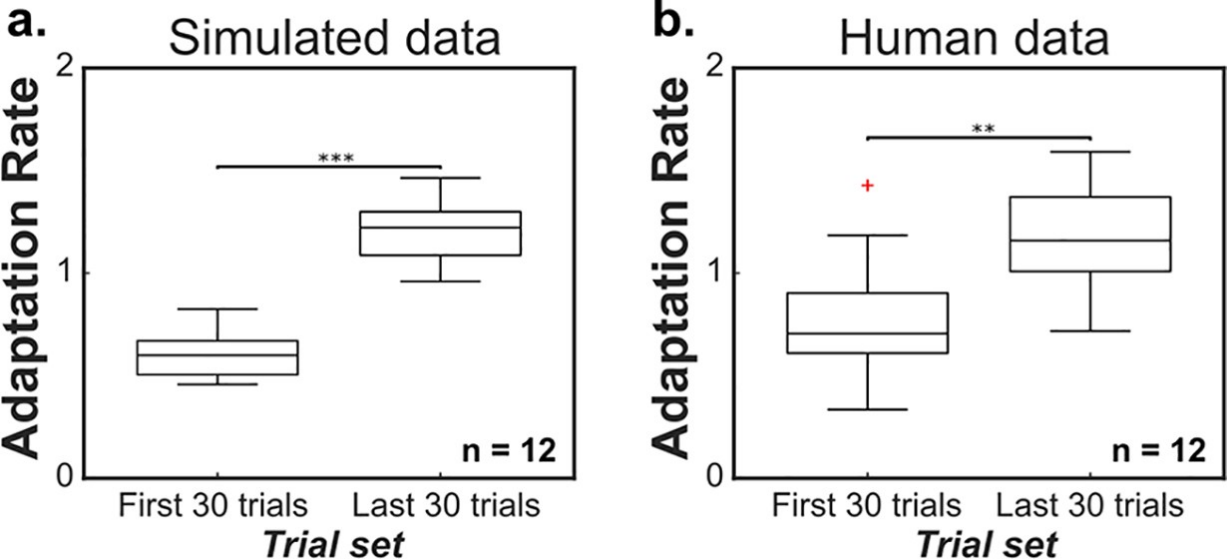
Adaptation rate depends on trial-set analyzed. Adaptation rate calculated from the last 30 trials of a 70-trial sequence of movements was significantly higher than the result from the first 30 trials for simulated experiments (a) and empirical data (b) (** = p<0.01, *** = p<0.001, two-tailed paired t-test).

We observed differences in regression coefficients when analyzing initial trials and steady-state trials (Fig 4). Even for the same system and the same sequential block of trials, different trial subsets resulted in statistically different adaptation rates (p<0.01, two-tailed paired t-test). These results were observed in data generated by the Bayesian learner model for a 12 subject simulation (p<0.001, two-tailed paired t-test; Fig 4A) and confirmed with analysis of human motor data (Fig 4B). All twelve human participants had a higher adaptation rate during steady-state trials compared to rates calculated from initial trials. Analysis of human data from another study [30] resulted in the same observed difference between trial-by-trial adaptation rates (S1 Fig). Results from a 10,000 subject simulation also resulted in a significantly higher adaptation rate for steady-state trials (μ = 1.13) than for initial trials (μ = 0.57; p<0.01, two-tailed paired t-test).

The main difference between initial and steady-state movement trials is the mismatch between the system’s parameters and the person’s estimate of those parameters. In our experimental setup, the mouse sensitivity, which was set unexpectedly low, would be the major system parameter affecting the cursor’s movement. During initial trials, the participant would be learning the mouse sensitivity by observing sensory prediction errors. Initially the mouse sensitivity estimation would not match the actual mouse sensitivity resulting in movement errors. This parameter misestimation is slowly corrected as learning progresses. During steady-state trials, the parameter learning has stabilized as the difference between the mouse sensitivity and the user’s estimate of the mouse sensitivity is minimized. Steady-state conditions allow for the calculation of a trial-by-trial adaptation rate that is unbiased by parameter misestimation.

The theory and data suggest that steady-state adaptation rates should be more consistent than those calculated using initial movement trials. But the question remains, how does a researcher know when steady-state has been reached? Some may suggest that steady-state occurs when error is minimized. This may sometimes be true but there could be times when there is zero mean error but internal model uncertainty, i.e. P_prm_ in the model (see Eq 7 in Methods), is still high. We next sought to develop a way to identify steady-state trials objectively in order to calculate trial-by-trial adaptation rates that were not corrupted by large parameter updates during early movement trials.

**Fig 5:**
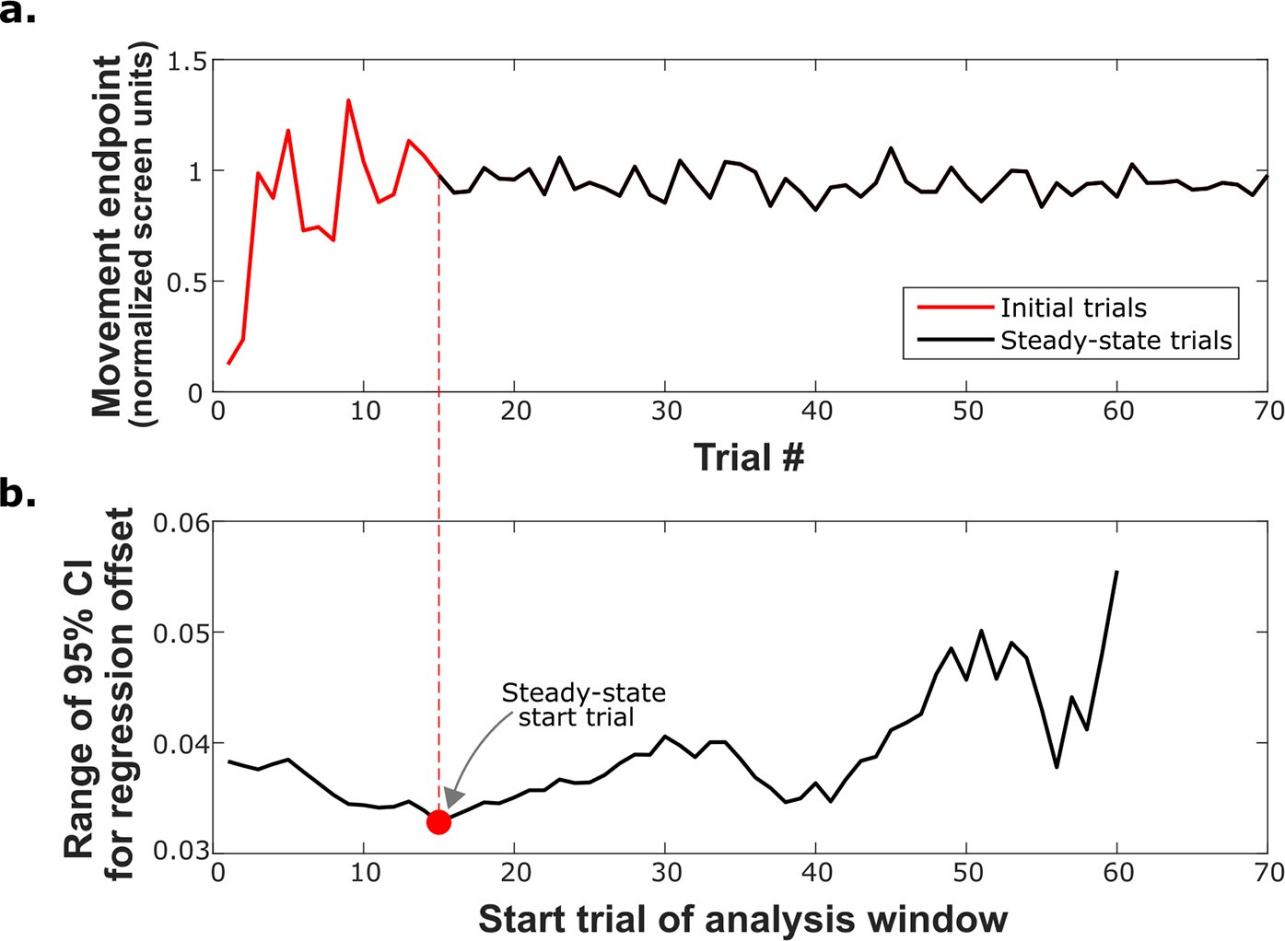
Novel protocol to identify steady state trials for adaptation analysis. (a) The expanding window protocol can identify the steady-state trials from a set of human movement data. (b) The start of the steady state trial set (which always ends at the last trial) is calculated as the point where the range of the 95% confidence interval of the offset of a zero-order robust linear regression is minimized. The minimum window size is 10 trials, thus the last potential starting trial plotted is trial number 60.

We developed a post-hoc analysis protocol that utilizes an expanding window technique to identify the steady state trials in a set of movement data (Fig 5A). Starting with the last ten trials of a person’s movement endpoint data—the smallest window suitable for trial-by-trial adaptation rate analysis—a robust linear regression is run with the first-order coefficient constrained to zero. The range of the 95% confidence interval of the offset is computed (essentially the robust mean). On the next iteration of the analysis, the window is expanded by one trial (i.e. to include the last 11 trials) and the offset confidence interval is recomputed. This is repeated with sequentially expanding windows until the last computation is run on a set of movement data that spans all trials. The trial window for which the confidence interval of the offset is minimized is identified as the steady-state trial set (Fig 5B). The trial-by-trial adaptation rate is then calculated using a robust linear regression across these trials. The standardized approach was able to identify a reasonable set of steady state trials across all human subjects (S2 Fig).

**Fig 6:**
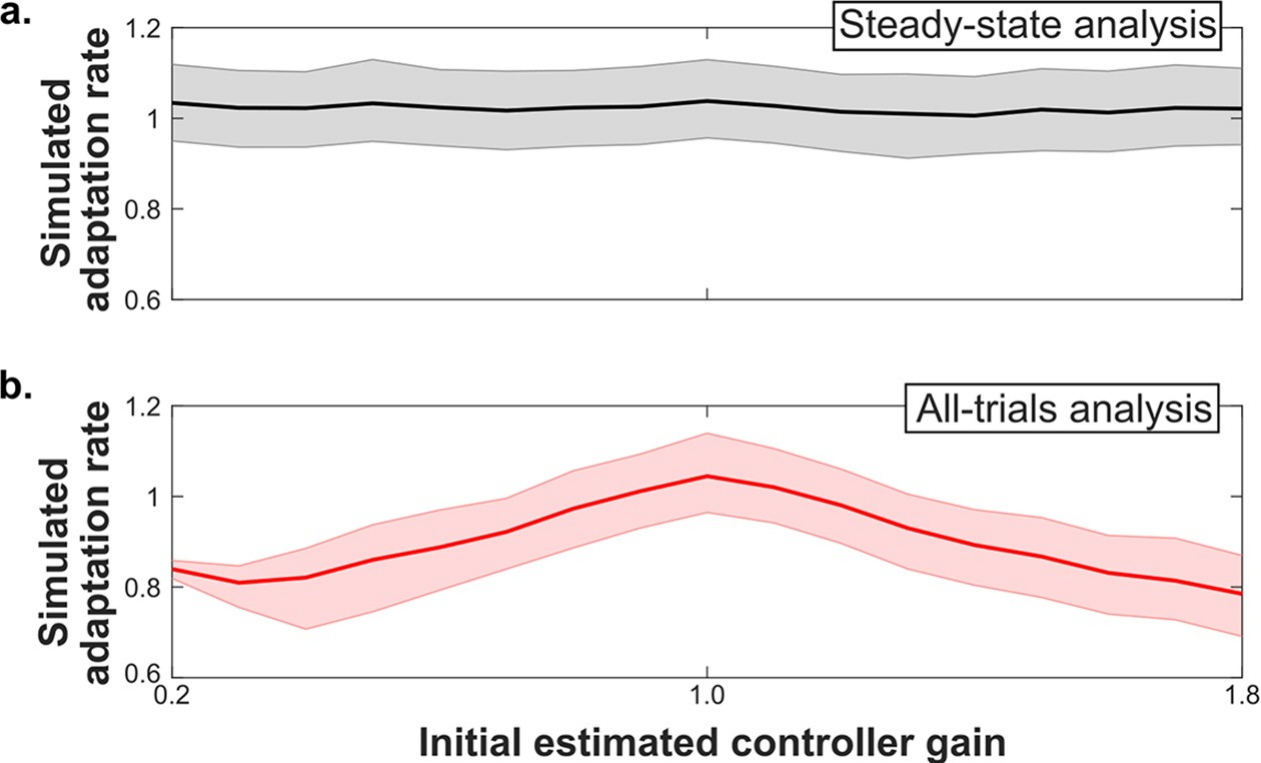
Steady state trial-by-trial adaptation rates show improved consistency. **(**a) As the initial gain estimate of the Bayesian learner model is varied, the resulting trial-by-trial adaptation rate calculated using the identified steady state trials remains consistent. (b) As the initial gain estimate is varied, the trial-by-trial adaptation rate calculated across all trials varies.

**Fig 7:**
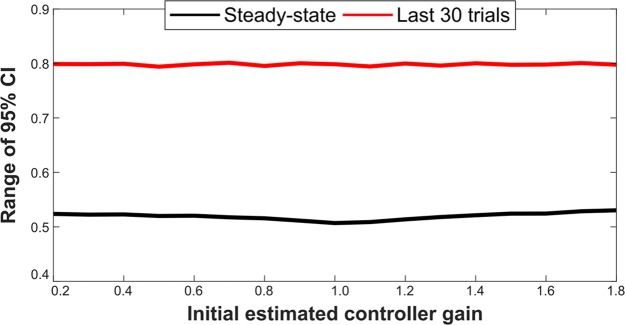
Steady state trial-by-trial adaptation rates show improved estimation confidence. The range of the 95% confidence interval of the regression coefficient representing the trial-by-trial adaptation rate is narrower when analyzing the identified steady-state trials compared to analysis of a constant number of trials (30) at the end of the experiment.

After developing the standardized protocol, we sought to compare the new approach with alternative analysis techniques. Using the Bayesian learner simulation, we simulated movement experiments with different initial parameter estimates. The learner was controlling a single degree of freedom movement with a single parameter: a control gain equivalent to the mouse sensitivity in the empirical study. We varied the initial controller gain and calculated the trial-by-trial adaptation rates across all trials, the last 30 trials, and the steady-state trials identified by the expanding window protocol (Fig 5). The novel approach results (Fig 6A) were more consistent than the analysis of all trials (Fig 6B) and similar to the analysis of the last 30 trials (S3 Fig). The range of the 95% confidence interval of the regression coefficient representing the trial-by-trial adaptation rate was lower for the steady-state trials than for analysis of the last 30 trials (Fig 7). Calculation of trial-by-trial adaptation rates from steady state trials produced more consistent and more confident estimates compared to the other trial sets analyzed.

With these results we have demonstrated a shortcoming of the conventional approach to calculating trial-by-trial adaptation rates. We first found that trial-by-trial adaptation rates are fundamentally distinct from perturbation adaptation rates. Then we provided evidence for a bias in trial-by-trial adaptation based on the trial set analyzed. We presented a novel analysis protocol that results in more consistent and more confident trial-by-trial adaptation rates. Our findings are predicted by a Bayesian learner model and supported by analysis of human movement data.

## Discussion

Here we have demonstrated with empirical and simulated data that the conventional approach to calculating trial-by-trial adaptation rates is biased. The dimensionless adaptation rate was previously considered to be an effective metric to compare results across experimental conditions, an assumption supported by static, single-layer models [19,31]. Here we show an inherent bias in the technique, results predicted in simulation using a hierarchical Kalman filter model describing human nervous system operation and confirmed with the analysis of human motor data. We showed that trial-by-trial adaptation rates are biased by the interacting effect of system learning. According to our Bayesian simulation results, initial system parameter misestimation had a major effect on the calculated adaptation rate. State-space only models [17, 19] fail to capture this characteristic of human motor planning and execution. Importantly, we propose a solution: a standardized analysis technique to objectively identify steady-state movement trials for more consistent and accurate results.

Empirical and simulation results show that the trial-by-trial adaptation rate is a biased metric. The overall learning (i.e. improvement of parameter estimation) in a trial-set of sequential movement trials affects the adaptation rate calculated across those trials. The empirical evidence is convincing, providing strong statistical support for this observation. A Bayesian simulation comprising a hierarchical Kalman filter model that has been supported empirically elsewhere [5] reproduces this adaptation rate bias observed between initial and steady-state trial analysis.

Results from the hierarchical Bayesian estimator suggest an explanation for this adaptation analysis bias: initial trial analysis suffers from the confounding influence of parameter misestimation. We suggest that trial-by-trial adaptation rates must be considered under steady-state conditions only, after the learning curve has reached an asymptote. Only steady-state adaptation rates are unaffected by parameter estimation errors and can be compared across subjects and experimental conditions.

Other factors not explored in this study may have affected the difference observed between initial and steady-state adaptation rates. For example, changing participant strategies or different weightings between explicit and implicit learning processes could have contributed to the observed differences [32]. We sought to reduce the effect of implicit learning processes by providing end-point only feedback in the human motor experiment [14].

Given the need to focus on steady-state trial-by-trial adaptation rates, we provide an expanding-window analysis technique to identify movements for which learning has stabilized. The approach identifies the trial set for which the confidence interval range of the estimate of the zero-slope robust regression offset coefficient is minimized. This confidence interval range will be lower when the variability of the data has stabilized but it also tends to decrease as more trials are analyzed. The result is a standardized estimate of steady state trials. By only considering adaptation rates from steady-state trials, meaningful comparisons can be made across subjects and experiments. The expanding-window approach to adaptation rate analysis represents a more consistent technique to be used by researchers in the field of human motor learning.

Differences observed between trial-by-trial adaptation rates and perturbation adaptation rates show that these are distinct quantities with different meanings. Trial-by-trial adaptation rates approach one under true steady-state conditions. Perturbation adaptation rates approach zero under true steady-state conditions. These two adaptation rates can both provide insight into a person’s motor performance but are quantitatively distinct and should not be compared to one another.

Moreover, trial-by-trial adaptation rates should not be used in perturbation studies. The perturbations reset the parameter estimation filter leading to post-perturbation trials with pronounced parameter misestimation. The trial-by-trial adaptation rate is affected by the initial trial bias and would not provide consistent results in perturbation studies.

One limitation of this study is that we assume a Bayesian model underlying movement generation. Recent evidence supports this assumption for many conditions [2,17] but there are exceptions [33]. The simulation results may be affected by the level of complexity of the model. A simpler state-space only model may be able to match the performance of the hierarchical Kalman filter model in certain conditions [19]. Alternatively, a broader model to capture the multiple time scales of learning driven by error history [12] or the recruitment of motor primitives driven by prospective error [34] could have been used. However, in steady-state conditions, the target for the analysis technique we propose, the slower learning processes have likely stabilized and the complexity of these models may not be necessary [12,34]. We also do not consider other learning processes such as use-dependent plasticity or operant conditioning [1]. Nevertheless, the hierarchical Kalman filter model seems to capture aspects of human motor performance at a level of detail that are relevant for clinical contexts.

Additional human experiments could be used to further test the Bayesian model adopted in this study. Although there exist empirical data of humans making movements with different levels of control and feedback noise [5,28], a careful study of this adaptation rate bias under these different noise scenarios would be insightful, especially to apply findings to neurorehabilitation contexts. We would expect more difficult motor tasks to be impacted even more by the initial vs. steady-state trial bias of trial-by-trial adaptation rate analysis. This hypothesis should be tested. Additional empirical work should aim to expand our understanding of human motor performance while also assessing the validity of Bayesian motor control models.

Ultimately this work has important applications in the clinical assessment of human movements. By more precisely measuring motor performance, without bias, we can make more accurate descriptions of human motor planning and execution. Improving adaptation rate analysis and interpretation is important for the field of movement neuroscience. The improved adaptation rate analysis we have presented will help advance basic motor control understanding that can directly impact the development of motor assessments [35], clinical rehabilitation techniques [36], and even biomimetic robots [37].

## Methods

### Ethics statement

Research with human subjects was completed with the approval and oversight of the University of New Brunswick’s Research Ethics Board and the U.S. Department of the Navy’s Human Research Protection Program.

### Subject recruitment

Twelve able-bodied right-handed volunteer study participants [mean age = 26.75yrs, range = 21-49yrs, 7 females] were recruited by word-of-mouth. Written informed consent was obtained from each participant before completing the experiment.

### Experimental setup

Hardware and mouse sensitivity settings have been reported elsewhere [26]. Subjects used a mouse with reduced sensitivity to move a cursor on the left side of a screen, constrained along a horizontal line, to land on a target on the right side of the screen. Endpoint only cursor position feedback was provided: the cursor disappeared after movement onset and reappeared at the end of the movement. Participants moved at their own pace and the movement distance was two-thirds of the lateral span of the screen (on-screen distance of 34.6 cm or 1282 pixels). All participants were naïve to the task and the mouse sensitivity settings. Each participant completed a block of 70 sequential movement trials. Testing lasted about 5–7 minutes.

### Simulations

All simulations were run in MATLAB. Each simulated experiment consisted of 70 movement trials, matching the empirical experiment length. All data are plotted as medians with error bars representing the 25–75% interquartile range, unless specified otherwise.

### Data generation

A hierarchical Kalman filter model [5,17] was used to generate simulated movement data. The movement endpoint position on the first simulated trial was calculated as

$$x_{\left[1\right]}=\frac{x^{\prime }*g}{{\hat{g}}_{\left[1\right]}}+E,$$ [1]

where *x*_*1*_ is the actual endpoint position on trial 1, *x′* is the target distance, *g* is the gain of the controller, ${\hat{g}}_{1}$ is the estimated gain of the controller on trial 1, and *E* is a noise term calculated from a Gaussian random number with a mean of zero and a variance of *Q*. The controller gain in the mouse cursor study is equivalent to the mouse sensitivity. In the catch-trial perturbation data we analyzed [25,38], the controller gain is equivalent to the EMG control gain.

A sensory observation, *z*_[1]_, was then calculated as

$$z_{\left[1\right]}=x_{\left[1\right]}+V,$$ [2]

where V is a noise term calculated from a Gaussian random number with a mean of zero and a variance of R.

The user’s perception of where they land, ${\hat{x}}_{\left[1\right]}$, will be a fusion between their expected value (x*′*) and their sensory observation (*z*_[1]_), and may be represented as a state-space Kalman filter update as

$${\hat{x}}_{\left[1\right]}=x^{\prime }+K_{S}(z_{\left[1\right]}-x^{\prime }),$$ [3]

where *K*_*s*_ is the state-space Kalman gain. The Kalman gain, constrained to a value between zero and one, is calculated for the single time-step endpoint-only feedback conditions as

$$K_{S}=\frac{Q}{Q+R},$$ [4]

which assumes that the state estimate uncertainty is equivalent to the controller variance, Q, due to a forgetting factor of one, and that the observation matrix is equal to one.

For each trial after the first, the estimated gain was updated in response to the error between where the user was trying to land (x*′*) and where they perceived that they landed (${\hat{x}}_{i}$) as

$${\hat{g}}_{\left[i+1\right]}={\hat{g}}_{\left[i\right]}+K_{P}({\hat{x}}_{\left[i\right]}-x^{\prime }),$$ [5]

where *i* is the trial number and *K*_*p*_ is the Kalman gain of the parameter estimation filter. The parameter estimation Kalman gain is iteratively updated as

$$K_{p[i+1]}=\frac{P_{prm[i+1]}}{P_{prm[i+1]}+R_{prm}},$$ [6]

where *R*_*prm*_ is the sum of *Q* and *R*, and *P*_*prm*_ is updated as

$$P_{prm[i+1]}=P_{prm[i]}(I-K_{p[i]})+Q_{prm[i+1]},$$ [7]

where *Qprm*, the trial’s parameter uncertainty, is calculated as

$$Q_{prm[i+1]}=\frac{{{(x}_{\left[i\right]}-x^{\prime })}^{2}}{P_{prm[i]}+R_{prm}}.$$ [8]

In light of the updated gain from Eq 5, the user’s subsequent endpoint position (*x*), sensory observation (z) and perception of endpoint position ($\hat{x}$) are simulated as was done for the first trial with Eqs 1–3 using the generalized equations

$$x_{\left[i+1\right]}=\frac{x^{\prime }*g}{{\hat{g}}_{\left[i+1\right]}}+E,$$ [9]

$$z_{\left[i+1\right]}=x_{\left[i+1\right]}+V,$$ [10]

$${\hat{x}}_{\left[i+1\right]}=x^{\prime }+K_{S}(z_{\left[i+1\right]}-x^{\prime }).$$ [11]

### Parameter settings

For the systematic testing of different initial controller gain values (Figs 6 and 7, S3 Fig), all parameters were held constant except for those indicated in the text and figures. Constant parameter values for Figs 6 and 7 and S3 Fig were: Q = 0.01; R = 0.01; P_prm-initial_ = 0.01; x*′* = 1; gain = 1. Each plotted point in Figs 6 and 7 and S3 Fig represents the median of 1,000 experiments run for that set of initial parameter values. For Fig 2A, the parameter values were: Q = 0.05; R = 0.07; P_prm-initial_ = 0.02; x*′* = 1; gain = 1, initial gain estimate = 0.5.

### Adaptation–linear regression

Similar to other studies [3,5], we calculated the trial-by-trial adaptation rate as the first-order coefficient of a linear regression calculated as

$${error}_{i+1}-{error}_{i}=b_{0}+b_{1}*{error}_{i},$$ [12]

where *error*_*i*_ is the movement error on the *i*th trial, and *b*_1_ is the adaptation rate. *b*_0_ is the intercept and usually close to zero. Except in the demonstration of the regression approach in Fig 3, we use a robust regression to calculate trial-by-trial adaptation rates utilizing the ‘RobustOpts’ option in MATLAB’s *fitlm* function.

### Perturbation analysis

To analyze trial-by-trial adaptation in response to a perturbation, we calculated the adaptation rate on each contiguous string of trials following a perturbation. For each experiment, we report the mean across all subsets of analyzed trials. We compared these values to the results obtained from the same trial sets using a perturbation study regression comparing error versus perturbation level [2]. In the simulation data (Fig 2A), experiment length (80 trials) and perturbation frequency (one perturbation occurring on the 6^th^, 7^th^ or 8^th^ trial of every 8-trial block) were set to match those of the empirical data (Fig 2B) [25,38]. The simulation perturbation level was set at 50% of the movement distance implemented as a shift in the simulated movement’s endpoint position at Eq 1. Mathematically, these constant perturbations were added similarly to the Gaussian distributed control noise. Self-generated error was determined by the combined effect of control noise and parameter misestimation.

### Steady-state analysis

We estimated the steady-state trials (i.e. when parameter learning stabilized) from a set of movement data using an expanding window protocol. First, a robust linear regression was run to fit an offset to the sequential endpoint position data. The range of the confidence interval of the estimated offset was recorded (with the slope, or first-order coefficient, constrained to zero the offset is essentially the robust mean). The first window analyzed contained the last 10 movement trials. Ten was considered the smallest number of trials to be selected for calculating the trial-by-trial adaptation rate. The first regression was run again using the last 11 trials, then the last 12 trials, and so on until the window of analyzed trials spanned all movement trials. The trial window for which the 95% confidence interval of the offset was minimized was considered to be the best sample of steady-state trials. This is the trial set for which a horizontal line was the best fit of the movement data indicating that ongoing parameter learning was minimized. The adaptation rate was then calculated using this identified set of trials using the approach described previously (see the ‘Adaptation–linear regression’ section).

### Data and code

Empirical data displayed in Figs 1 and 2B are from Shehata et al. [25] and are available here: https://doi.org/10.5061/dryad.v12f25n [38]. Data in S1 Fig are from Ikegami et al. [30]. Empirical data in Figs 3, 4B, 5 and S2 were collected in this study. These data, analysis code and simulation code are available from the Dryad Digital Repository (https://doi.org/10.5061/dryad.vd561).

## Supporting information

S1 Appendix Mathematical derivation demonstrating the relationship between linear regression and autocorrelation analysis techniques. (DOCX)

S1 Fig Initial vs. steady state trial-by-trial adaptation rates for subjects from Ikegami et al. 2012 [31]. Same as Fig 4B but for a different dataset.
(PNG)

S2 Fig Steady-state trial identification from all 12 human subjects. Same as Fig 5A but for all subjects tested.
(PNG)

S3 Fig Steady state trial-by-trial adaptation rates using the last 30 trials. As the initial gain estimate of the Bayesian learner model is varied, the resulting trial-by-trial adaptation rate calculated using the last 30 trials remains consistent, similar to the steady-state trial analysis results in Fig 6A.
(PNG)
