# Supplementary material for: Conventional analysis of trial-by-trial adaptation is biased: Empirical and theoretical support using a Bayesian estimator
Source: PLoS Comput Biol. 2018 Dec 26;14(12):e1006501. doi: 10.1371/journal.pcbi.1006501 (PMC6324815; doi:10.1371/journal.pcbi.1006501)
Supplement: S1 Appendix — (DOCX) [file pcbi.1006501.s001.docx]

**Supporting information**

**S1 Appendix. Mathematical derivation demonstrating the relationship between linear regression and autocorrelation approaches.**

The linear regression and autocorrelation approaches to measuring motor adaptation are closely related as shown in this mathematical derivation.

Autocorrelation

The general expression for the autocorrelation function for a lag of $\tau$ samples is

$\Phi_{xx}\left( \tau\right)= \frac{E\left[ \left( x\left( t \right) \right)(x\left( t+\tau\right))) \right]}{\sigma_{x}^{2}}$, [S1]

where $\sigma_{x}^{2}$ is the variance of all movement endpoints, x(t) is the movement endpoint at trial t, and x(t+τ) is the movement endpoint at the τ^th^ trial following trial t (lag τ). In discrete time, the time index is expressed in samples: $x_{i}\equiv x(iT_{s})$, where $T_{s}$ is the sampling period, and the first lag is

$\Phi_{x}\left( 1 \right)= \frac{E\left[ \left( x_{i} \right)(x_{i+1}) \right]}{\sigma_{x}^{2}}$. [S2]

Since $cov\left( x_{i}{,x}_{i+1} \right)=E\left[ x_{i}x_{i+1} \right]-{\mu_{x}}^{2}$, we arrive at

$\Phi_{x}\left( 1 \right)=\frac{cov\left( x_{i},x_{i+1} \right)}{\sigma_{x}^{2}}+\frac{{\mu_{x}}^{2}}{\sigma_{x}^{2}}$, [S3]

where µ_x_ is the mean of all movement endpoints.

Linear Regression

The general expression for the linear regression is $Y=aX+b$ where *X* is used to predict *Y*. The regression analysis provides the slope *a* and the y-intercept *b.* This is usually solved using a mean-square error criterion. With this criterion, the solution may be expressed probabilistically as

$E\left[ Y | X \right]=aX+b$ [S4]

where $E\left[ Y | X \right]$ is the expected value of *Y* given *X.* The parameter of interest is *a* which is the regression slope. The mean square error solution for *a* is

$a=\frac{E\left[ X \right]E\left[ Y \right]- \mu_{X}\mu_{Y}}{\sigma_{X}^{2}}$ [S5]

Here, $X=Err_{i}$ and $Y=\Delta Err_{i}$ so that

$a=K_{r}=\frac{E\left[ Err_{i} \right]E\left[ \Delta Err_{i} \right]- \mu_{Err_{i}}\mu_{\Delta Err_{i}}}{\sigma_{Err_{i}}^{2}}$ [S6]

The product of the error means $\mu_{Err_{i}}\mu_{\Delta Err_{i}}$approaches zero for steady-state data, so we simplify *K_r_* as

$K_{r}=\frac{cov\left( Err,\Delta Err_{i} \right)}{\sigma_{Err}^{2}}=\frac{cov\left( Err_{i+1},Err_{i} \right)}{\sigma_{Err}^{2}}-\frac{cov\left( Err_{i},Err_{i} \right)}{\sigma_{Err}^{2}}=\frac{cov\left( Err_{i+1},Err_{i} \right)}{\sigma_{Err}^{2}}-1$ [S7]

which further simplifies to:

$K_{r}=\frac{cov\left( x_{i+1},x_{i} \right)}{\sigma_{x}^{2}}-1$ [S8]

Autocorrelation and linear regression comparison

Comparing Eq. S3 with Eq. S8, we see that the expressions for ACR(1) and *K_r_* for steady-state data differ only by a shift. We can summarize this relationship as

$K_{r}=ACR\left( 1 \right)-1-\frac{{\mu_{x}}^{2}}{\sigma_{x}^{2}}$. [S9]
