# Supplementary figures and images for: Conventional analysis of trial-by-trial adaptation is biased: Empirical and theoretical support using a Bayesian estimator
Source: PLoS Comput Biol. 2018 Dec 26;14(12):e1006501. doi: 10.1371/journal.pcbi.1006501 (PMC6324815; doi:10.1371/journal.pcbi.1006501)

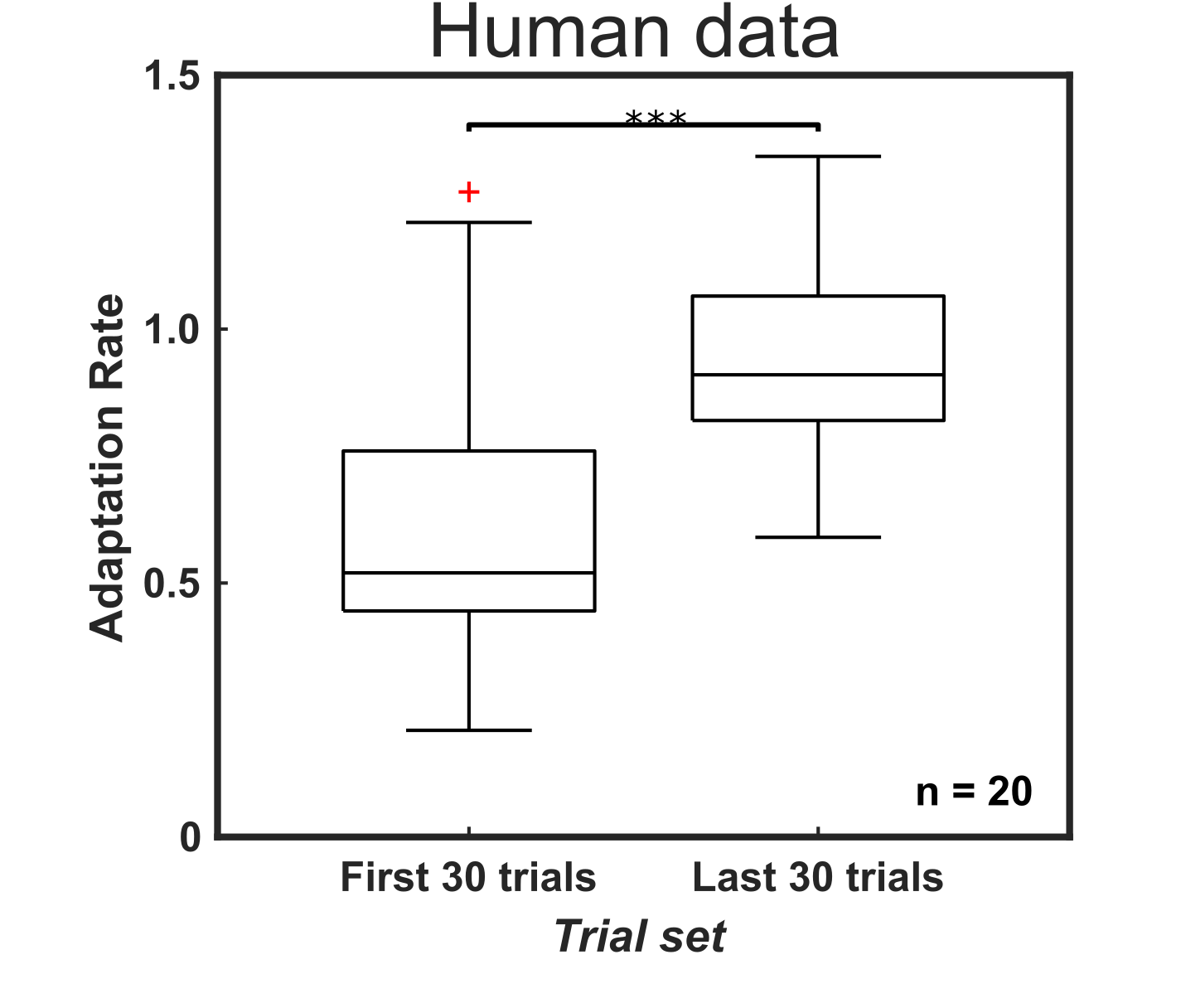

Supplement: S1 Fig — Same as Fig 4B but for a different dataset. (PNG) [file pcbi.1006501.s002.png]

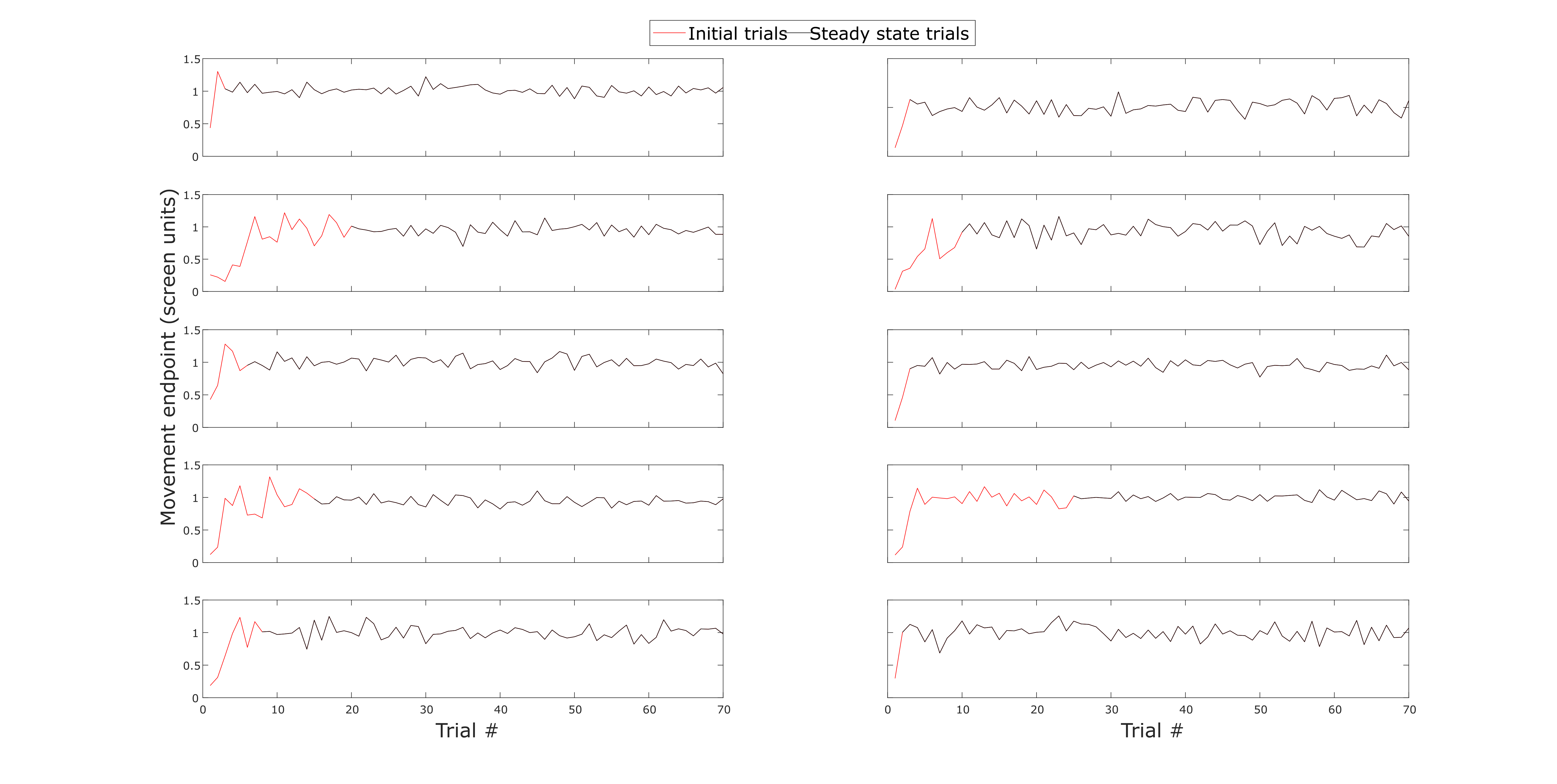

Supplement: S2 Fig — Same as Fig 5A but for all subjects tested. (PNG) [file pcbi.1006501.s003.png]

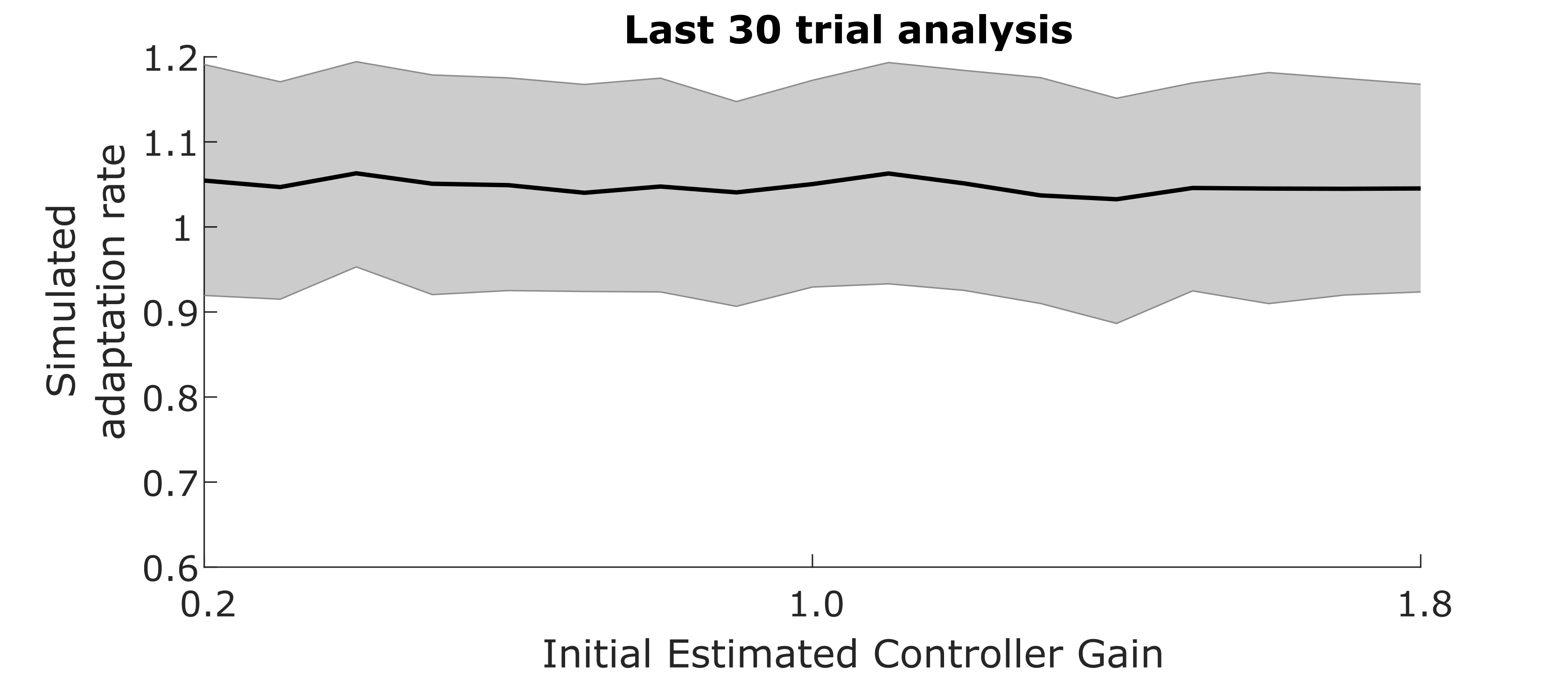

Supplement: S3 Fig — As the initial gain estimate of the Bayesian learner model is varied, the resulting trial-by-trial adaptation rate calculated using the last 30 trials remains consistent, similar to the steady-state trial analysis results in Fig 6A. (PNG) [file pcbi.1006501.s004.png]
